# National Vulnerability to Pandemics: The Role of Macroenvironmental Factors in COVID-19 Evolution

**DOI:** 10.1155/2022/9524407

**Published:** 2022-03-24

**Authors:** Muhammad Aljukhadar

**Affiliations:** HEC Montreal, Department of Marketing, Office No. 4.501, 3000, Chemin de la Côte-Sainte-Catherine, Montréal, Québec H3T 2A7, Canada

## Abstract

The aim of this research is to show that vulnerability to pandemics is unequal across nations, and that culture besides other national factors helps unleash some of the disparities. A nation's vulnerability to pandemics is defined as a state of fragility and dereliction at the national level perceptible at the early stage of the emergence of a pathogen when no definite information is available about it and no clear response is in place, creating a dependence on national factors as well as contextual factors. That is, vulnerability reflects the evolution or spread of a nascent pandemic in a given nation. A set of hypotheses that prescribe how a nation's factors would contribute to its vulnerability is developed. Data reflecting the national factors of a sample of countries that reported early COVID-19 cases were collected from secondary sources to test the hypotheses. The results show that, whereas factors such as economy and healthcare had a modest effect, two cultural factors were salient in shaping a nation's vulnerability to COVID-19. Furthermore, poverty prevalence associated with a nation's vulnerability. Delineating how a nation's culture and macroenvironmental factors shape its vulnerability at early stages of pandemic evolution, the results encourage policymakers to extend timely support to nations high on uncertainty avoidance and low on indulgence, as well as where poverty is prevalent. Such nations require proactive measures such as behavioral interventions and communications that are culturally sensitive and inclusive.

## 1. National Vulnerability as a Factor to Manage the Evolution of Nascent Epidemics

Well before the COVID-19 outbreak, the Global Health Risk Framework (GHRF) Commission indicated that pandemics cost the world US$ 60 billion annually and underscored how eminent is their threat to global security and consumer welfare ([1]; for details on the GHRF, see https://nam.edu/initiatives/global-health-risk-framework/). In the last twenty years, we witnessed many outbreaks and pandemics, stressing the need for a better response at the national and international level. To manage pandemics effectively, policymakers face a myriad of challenges including financial preparedness, response efforts coordination, securing human and material resources, and reinforcing prevention and control measures while balancing the investments [2]. For an optimal allocation of investments, and to improve wellbeing at large, scholars need to identify the consumer groups disproportionately vulnerable to pandemics. Scholars have so far focused on vulnerability and risk factors at the individual level, which show the relevance to study vulnerability at the nation level. Indeed, “vulnerability to the COVID-19 pandemic cannot be fully explained by individual risks alone but rather by broader social and structural determinants of health that result in inequities in communities where vulnerable populations live, work, play, pray, and learn” ([3], pg. 1–2).

Scholars have coined the term social vulnerability to envisage the resilience of communities to various health threats [4]. Applying the concept to fathom pandemic evolution is needed. While recent research adopted the concept, it approached vulnerability from a risk-exposure lens, relating it to morbidity and fatality in a given nation [5]. Starting from the literature and Baker et al. [6] definition of consumer vulnerability, a nation's vulnerability to pandemics is viewed herein as *a state of fragility and dereliction at the national level perceptible at the early stage of the introduction of a pathogen when no definite information is available about it and no clear response (crisis management program, confinement policy, etc.) is in place, creating a dependence on national factors (demography, economy, healthcare, geography, and culture) and contextual factors (season, international aid, and pathogen characteristics such as infection and morbidity rates)*. In other words, vulnerability reflects the extent to which a nation would suffer from the evolution of a nascent pandemic, thus contributing to its spread. Vulnerability to pandemics is at play at both levels—the individual and the national. For instance, a country with a better healthcare system should be less vulnerable at the national level, fueling lower vulnerability for the individual. Likewise, a country with cultural norms and practices that counter pathogen spread will be less vulnerable, fueling lower individual vulnerability and better welfare for everyone. To inform policymakers, the focus herein will be vulnerability at the national level. For consistency, a nation's vulnerability to pandemics is termed vulnerability hereafter.

It is argued that national culture, besides other macrofactors, shapes vulnerability. Culture is the shared values, norms, and codes that shape the beliefs, attitudes, and behavior of a group of people through their interaction with themselves and with the environment; indeed, investigating how culture affects health helps see the forest rather than only the trees [7]. Focusing on the role of culture is relevant because “lack of cultural and contextual knowledge can maintain cultural barriers in the communities we serve and hinder measures to contain and eventually respond to the pandemic. Therefore, the cultural concepts in developing strategy and communication are important.” [8]. Given that culture is understudied in healthcare and health policy research, scholars have adapted cultural models to show the central role of culture in explaining health beliefs and outcomes. The PEN-3 model for instance contemplates the cultural effect as it embodies people's identity, relationship, and empowerment [9]. These models show the key role of culture to develop and evaluate health and wellbeing policies, displaying how culture acts as a social web that shapes people's judgments and behaviors regarding health, healthcare, and wellbeing in general [10].

Scholars have grouped individuals according to their beliefs and values. In a variety of contexts, scholars used national culture to study people vulnerabilities. For instance, the cultural dimension of uncertainty avoidance positively influences people's financial literacy, while individualism negatively influences it [11]. Recently, Akaliyski et al. [12] showed the validity of the national culture as a collective phenomenon that supersedes individual differences and other social aggregates. Cultural practices and people wellbeing are intertwined. People in collectivistic countries (where pathogen prevalence is higher compared with individualistic countries) show more conformity, ethnocentrism, and other collectivist-related behaviors that help control pathogen spread [13]. The purpose of this research is to underscore the role of a nation's culture and other macro factors in explaining its vulnerability to the COVID-19 pandemic.

## 2. Literature and Hypotheses

Several countries (Thailand, Australia, Hong Kong, Singapore, Malaysia, Kuwait, and the US) were early at announcing COVID-19 cases [14, 15]. However, they showed initial resistance toward it, or low vulnerability, compared with the four countries Italy, Iran, Japan, and South Korea who have been disproportionally and severely affected by the nascent epidemic during the same period—showing high vulnerability [14, 15].

Why these four countries, IIJS hereafter, were severely affected, reporting early COVID-19 outbreaks? Arguably, IIJS have sustained COVID-19 evolution outside its origin country until March 13, 2020, when the WHO declared it a global pandemic. Shortly after that date, a new phase started with the European countries of Spain, France, and Germany reporting the majority of new cases. Anderson et al. [16] suggested that IIJS acted as a central incubator that turned the malady into a global pandemic, affecting the wellbeing of consumers at large. Had IIJS showed low vulnerability to COVID-19, as the other seven countries had, the epidemic evolution would have followed a different path. According to the provided definition of vulnerability, this research will focus on the COVID-19 early outbreak, i.e., from its appearance until when declared a pandemic. It aims to examine the role of a nation's macro factors including culture in shaping national vulnerability to the novel coronavirus.

National culture, besides other relevant factors, should shape the national vulnerability. Rathod [8] indicates, “Culture plays a central role in determining attributions to illness, help seeking behaviours and pathways, and community willingness to comply with measures to counter a pandemic spread. Therefore, cultural beliefs and values can contribute to the success or failure of global efforts to contain spread of an outbreak.” The history is abundant in supporting exemplars, e.g., cultural practices such as burial have contributed to Ebola evolution [17]. Understanding culture is a first step to fight epidemics [18].

Scholars have contemplated the role of culture in the context of epidemics and healthcare. Ji et al. [19] studied how culture influences the spread of a previous acute respiratory epidemic (SARS). They found that Chinese consumers show harmful optimism (they believed the odds of contracting SARS themselves are lower than that of their friends) and lower pessimism than Canadians. These authors also found that Chinese consumers were more able to embrace the positive changes imposed by SARS. Uskul [20] discussed how culture shapes various aspects of health, such as adapting to pain, seeking healthcare assistance, and regulating the relation with doctors and other healthcare providers. The author then conferred how a health communication that adapts to the cultural context would have a higher level of persuasion. Betsch et al. [21] studied how culture influences people's acceptance of vaccine. They found that countries with collective cultures are more accepting of vaccines; they thus recommended extensive prosocial nudging in individualistic countries to increase the vaccination rate.

Recently, scholars have showed the role of culture in the context of COVID-19. Ibanez and Sisodia [22] discussed the adequacy of Hofstede's cultural factors to understand COVID-19 adverse impacts. They found that a nation's individualism, power distance, masculinity, long-term orientation, and indulgence, along with economic performance, shape the COVID-19 death ratio. Focusing on the death caused by COVID-19, Güss and Tuason [23] found that, even when controlling for national factors such as GDP, population density, age, BMI, and the Gini index, countries high on power distance and collectivism enjoyed lower COVID-19 fatality. They conclude that “group-oriented and collectivist values and low egalitarianism values” contributed favorably to COVID-19 fatality. Following a cross-cultural Bayesian analysis, Kano Glückstad et al. [24] found that cultural values shape healthcare indicators depending on degree of local infections; pleasure seeking differently modulates the anxiety toward infection according to culture. Their results underscore, “… the importance of considering both cultural and individual contexts when policymakers are going to develop measures to address pandemic dilemmas such as maintaining public health awareness…” Focusing on the first COVID-19 wave, Erman and Medeiros [25] found that uncertainty avoidance and long-term orientation of a nation is associated with a higher COVID-19 fatality rate. Windsor et al. [26] indicated that, when both individualism and power distance are high in a country, the average COVID-19 death rate was very high (28.79 per 100,000); when both cultural factors were low such as for New Zealand and Trinidad and Tobago, the death rate was much lower (1.89 per 100,000). Using the GLOBE model, and focusing on COVID-19 morbidity and mortality, Kumar [27] found a favorable role for power distance, institutional collectivism (rather than group collectivism), and performance orientation.

While these recent studies show the relevance of considering national culture, they focus on the consequence of COVID-19 (morbidity and/or mortality) over extended periods. In addition, the recent studies do not show a prominent role for economy, healthcare, and other development indexes in limiting COVID-19 consequence. On the contrary, countries with the highest economic development showed the highest COVID-19 mortality. Williams et al. [28] tried to explain this paradoxical trend, citing reasons such as citizens' ability to travel thus more infections.

Alternatively, this paper focuses on studying COVID-19 evolution—embodied in a nation's vulnerability—using a macro lens. In fact, the inconclusive results in the recent studies and their focus on the COVID-19 death ratio favor the concept of vulnerability—studying a pandemic evolution at its early phase across nations. Like the majority of recent studies, this paper examines national culture according to Hofstede's theory [29]. When a pathogen such as the 2020 SARS-CoV-2 emerges, citizens of a nation should enjoy certain cultural values and practice to limit its initial spread, i.e., a low-vulnerability nation. For example, nations where the majority of citizens exhibit an adequate level of collaboration, awareness, and responsiveness to early warnings communicated about COVID-19 would show low vulnerability. Likewise, nations where the majority of citizens single-handedly adhere to advice and authorities' instructions would show low vulnerability. That is, nations should enjoy certain prevalent cultural codes and behaviors (and citizens should be adaptable to challenges and stressors) to enjoy a low vulnerability to emerging pandemics.

National culture encompasses such codes and behaviors [11, 12]. Hofstede [29] indicates that nations differ on six dimensions: individualism-collectivism (the assimilation of individuals into principal groups), uncertainty avoidance (the level of stress facing an unknown future), power distance (strength of social hierarchy), masculinity-femininity (the allocation of emotional roles between men and women), long-term orientation (individual focus on the future, or the present and past), and indulgence versus self-restraint (individual control of desires related to enjoying life). Tausch [30] recently revalidated Hofstede's dimensions, finding ample support for four of them—power distance, individualism, indulgence, and long-term orientation.

The following discusses Hofstede's dimensions [31] and underlines how they shape a nation's vulnerability:

- Uncertainty avoidance predicts how a society faces stress under unclear situations. When this factor is low, a society deals with unknown and confusing situations in a good way; cultural practices in the case of a mysterious and infectious malady would readily entice hygiene, social distancing, self-quarantine, and confirming with confinement instructions. Therefore, low uncertainty avoidance reduces vulnerability.
- Indulgence predicts lower self-control, higher gratification, and life satisfaction. Restrain societies show high control over cravings and desires, strong compliance to strict social norms, and high nihilism and cynicism. At an early stage of an epidemic, when no confinement policy is present, a society with high indulgence is better able to respond. Therefore, a high indulgence reduces a nation's vulnerability.
- Long-term orientation predicts whether a society relays on its past and prospect. When this factor is high, a nation would be prepared, e.g., has a crisis management system in place. Therefore, a high long-term orientation reduces vulnerability.
- Individualism predicts a slack social group where a person takes care of oneself and direct family. A society low on this factor (a collective society) is better at responding to epidemics as shown in prior work. Therefore, a low individualism reduces a nation's vulnerability.
- Power distance predicts whether people accept the unequal distribution of power within family and other social systems. During the early stage of a pandemic, a nation low on power distance would be better off as consumers do not require a clear confinement policy and control measures to behave responsibly. Therefore, a low power distance reduces vulnerability.
- Masculinity predicts rivalry, decisiveness, and heroism. A nation low on this factor encourages cooperation and caring for others. Therefore, a low masculinity reduces vulnerability. The author observed a negative influence of masculinity, specifically the macho identity; during discussions with citizens in a region high on masculinity, the explanation usually cited for not wearing masks nor keeping social distance was believed that “real men do not do that.”

These propositions are similar to the ones reported recently by Windsor et al. ([26], p. 7). These authors studied the impact of culture on the COVID-19 death rate and hypothesized, “We expect that a particular combination of cultural traits would support better outcomes during a pandemic …: more indulgence; less uncertainty avoidance; more collectivism; more long-term orientation; more femininity; and less power distance in society.” Therefore:

**Hypothesis 1 .**

A nation's cultural factors associate with its vulnerability to pandemics such that vulnerability is low when **(a)** uncertainty avoidance is lower, **(b)** indulgence is higher, **(c)** long-term orientation is higher, **(d)** individualism is lower, **(e)** power distance is lower, and **(f)** masculinity is lower.

Other national factors might as well determine vulnerability. Factors characterizing a nation such as demographics, geography, economy, and healthcare would determine vulnerability, as the theory proposes (for **H2-**specific hypotheses and their results, see Table 1). For instance, Ibanez and Sisodia [22] showed that economic performance shaped the COVID-19 death ratio. Similar results are found by Grima et al. [5], who developed a pandemic risk exposure measure using national morbidity and mortality as dependent variables. Therefore:

**Hypothesis 2 .**

A nation's demographics, geography, economy, and healthcare factors associate with its vulnerability to pandemics.

## 3. Methodology and Results

To investigate the hypotheses, the case study approach was firstly followed. Since the first incidence of COVID-19 was reported in China's Wuhan province in the late 2019 and until the WHO declared it a pandemic, IIJS have been disproportionally fast in spreading it. By then, IIJS had far more cases than the rest of the world. According to Anderson et al. [16], one can argue that Italy facilitated the novel coronavirus spread to European neighbors, whereas South Korea and Japan facilitated its spread to Southeast Asia, US, and Australia. Likewise, Iran facilitated its spread to Russia, Arabian Gulf, Syria, and Lebanon given strong ties with these countries. In summary, COVID-19 evolution in IIJS during that early phase has contributed to its steep evolution. March 13, 2020, was considered the end to COVID-19 evolution because the WHO then announced it a pandemic. Starting from this date, policymakers and consumers more properly recognized the repercussions associated with the nascent pandemic [32], and the role of culture in determining vulnerability would dissipate. Indeed, mass communication of preventive orders and confinement measures imposed by policymakers amplified after WHO announcement.

Secondly, the demographics, geography, economy, and healthcare factors for the nations that were early at announcing COVID-19 cases (i.e., IIJS, Thailand, Australia, Hong Kong, Singapore, Malaysia, Kuwait, and the US) were inspected. Following a comparative analysis, the author examined the propositions implied by **H2**, i.e., factors such as socioeconomics influence vulnerability. One would expect that vulnerability would be low for a nation with a versatile and universal healthcare system, higher per capita income, and better economy indicators [33]. The comparative analysis showed that, in the COVID-19 case, such factors were not salient in influencing vulnerability. IIJS diverge on these factors. While Iran lacks on healthcare, Italy, Japan, and South Korea each has a notable healthcare system. In addition, per capita income is higher for the latter countries. Then, I focused on the role of culture and noted that two factors were salient at influencing vulnerability: uncertainty avoidance and indulgence. Nations that early-reported COVID-19 cases but showed low vulnerability (Thailand, Australia, Hong Kong, Singapore, Malaysia, Kuwait, and the US; [14]) are generally low on uncertainty avoidance and high on indulgence. Meanwhile, IIJS (which showed large chains resulting in extensive COVID-19 evolution; [14]) are generally high on uncertainty avoidance and low on indulgence, i.e., nations of restraint.

**Figure 1:**
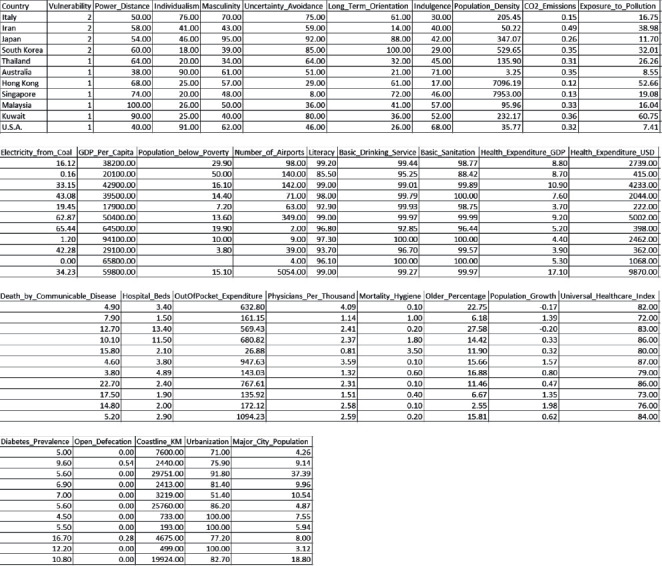
Research secondary data.

To verify these observations empirically, the author compiled a data set comprising a nation's score on Hofstede's cultural dimension (from Hofstede-Insights.com) and relevant national factors (from the WHO, World Bank, and IndexMundi that report a nation's socioeconomic and related factors).  Figure 1 reports the compiled data used in the analysis.

To test **H1**, which states that a nation's cultural factors influence its vulnerability such that vulnerability is low when (a) uncertainty avoidance is lower, (b) indulgence is higher, (c) long-term orientation is higher, (d) individualism is lower, (e) power distance is lower, and (f) masculinity is lower, bivariate correlation was performed (Table 2). The results lend support to **H1a** and **H1b**.

Similarly, to test **H2**, bivariate correlation was performed (Table 1 for *p* value of correlation coefficient). Consequently, one hypothesis (**H2b**) was supported. Furthermore, three hypotheses (**H2a**, **H2n**, and **H2o**) were marginally supported, i.e., supported at the 10% significance level. Given the small sample size (11 countries or observations), the latter three hypotheses are discussed next.

## 4. Discussion and Implications

The findings of **H1** show that two cultural factors relate to a nation's vulnerability to pandemics. Firstly, nations high on uncertainty avoidance showed high vulnerability. When this dimension is low, a nation deals with unknown and ambiguous situations in a better way, and cultural practices in the case of a mysterious and infectious malady would encourage hygiene, social distancing, self-quarantine, and confirming with initial confinement policy and instructions. South Korea and Japan are among the most uncertainty-avoiding nations in the world.

Nations high on uncertainty avoidance show rigid codes of belief and behavior and are intolerant of unorthodox ideas and behaviors [31]. They tend to rely on divine power and authority figures representing such power. These figures usually instill hopes and promise protection in unlawful manner. Reports documented that consumers engaged in unsafe group practices to stop the COVID-19 malady, such as bathing in animal dung (Southern Asia), drinking lethal ethanol (Iran), and eating certain foods like garlic. To enjoy lower vulnerability, consumers had to adapt their behaviors swiftly, which is less feasible in uncertainty-avoiding nations. In these nations instead, there is a need for rules, time is money, consumers are workaholic, punctual, and precise while they tend to discard innovations [31].

Meeuwesen et al. [34] studied rapport building between physicians and patients in various nations, concluding “the higher the level of uncertainty avoidance, the less the attention is given to rapport building.” This finding was replicated for prosocial behavior [35]. The rapport between physicians and patients are determinant of vulnerability to pandemics, rather than the sheer number of physicians per capita. Such practices and predispositions contribute to high vulnerability.

Nations low on uncertainty avoidance have shown low vulnerability. In the US and other nations low on this dimension, context drives people to reshape their behaviors continually—there is freedom of expression, acceptance for new concepts, and willingness to try different experiences and products [36]. In addition, uncertainty avoidance leads to inferior governance. Nations high on uncertainty avoidance tend to have incompetent governmental policies [37].

Secondly, nations low on indulgence showed high vulnerability. These nations have societies of restraint where consumers have a tendency to cynicism and pessimism [31]. This tendency drives consumers to underestimate threats and abide less with initial confinement and hygiene instructions. Consumers in these nations tend to control the gratification of desires and assign less importance to leisure time, and social norms are more influential. Resisting social norms (stopping family visits, committing to social distancing, putting on a facemask in the company of friends, etc.) is thus challenging. These practices make the nations low on indulgence more vulnerable to pandemics. During the Ebola spread, culturally unaware messages such as “Don't touch anyone that contracted Ebola” were ineffective; consumers do not conform to such messages due to strong social norms.

At its early phase, COVID-19 implicated a high level of uncertainty and confusion, such as symptoms and changing policies [16]. A nation's vulnerability thus primarily relates to the people ability to mitigate uncertainty and adapt. The empirical results advance the notion that two cultural factors (uncertainty avoidance and indulgence) shape vulnerability. These two factors also help predict the countries with the highest vulnerability in the following week, i.e., between 13 and 20 March, 2020; the three countries with the most reported COVID-19 cases were Spain, France, and Germany [14]. These three countries, which made Europe the epicenter in that period, are high on uncertainty avoidance and low on indulgence. Citizens' approval of government COVID-19 policies considerably differ even across countries from the same region, i.e., EU [38]. Travica [39] argues that culture shapes COVID-19 spread because culture influences confinement response and policy. At an advanced phase (more than two weeks after declaring COVID-19 a pandemic), the role of national culture notably dissipates, and almost all countries in the world started reporting significant increments in COVID-19 cases. By then, almost all countries imposed reactive measures comprising full or partial lockdown, social distancing, hygiene codes, and borders control or closure. It is not surprising that research shows that the sociodemographics of people infected during the first wave differ a lot from those of people infected at a later wave [40].

The findings of **H2** show that certain national factors influence vulnerability. Similar to recent research linking GDP to COVID-19 death ratio [25], GDP per capita marginally and negatively correlates with vulnerability. Interestingly, the results show that the percentage of population below poverty line significantly correlates with vulnerability. That is, from the socioeconomic factors studied, poverty prevalence in a nation was a determinant factor of vulnerability. Poverty indicates that more people are less educated, have less or no access to healthcare, live in slums, and more prone to erroneous health practices and beliefs. These elements fuel a nation's vulnerability to pandemics.

The second finding was that vulnerability correlates with the number of hospital beds per 1,000 people (positive and significant correlation), annual population growth (negative and marginal correlation), and the percentage of population 65-year-old or more (positive and marginal correlation). This finding, similar to that of Williams et al. [28], implies that vulnerability increases for nations with aging populations. Such nations are usually industrialized, have lower population growth, spends more on healthcare, and have more hospital beds.

The majority of the national factors tested in **H2** did not correlate with vulnerability, suggesting that culture has a prominent role. Indeed, a nation's vulnerability would “…more likely to be directly driven by socio-cultural factors that represent the baseline behaviors as well as the immediate behavioral shifts or reactions to such a situation in contrast to economic concerns, which likely play a relatively greater role in shaping the responses and outcomes during the subsequent and more prolonged stages of the pandemic.” [25]. The findings suggest that the COVID-19 epidemic acted as a stressor causing health, wellbeing, and economic anxieties, and some nations were better at initially resisting it given proper cultural values and practices.

## 5. Conclusions and Recommendations

Before the WHO declared COVID-19 a pandemic, the information communicated about this malady was tentative and indeterminate. For one, the flow of information from Wuhan city to China as well as the rest of the world was asymmetric. In addition, many political and opinion leaders downplayed its risks. Consumer cultural values and responsiveness are influential during the early phase of pandemics, i.e., before policymakers and authoritative figures weigh in and before sufficient information about the malady is available. The national culture captures consumer awareness, responsiveness, and predispositions. Cultural factors shape whether consumers comply with socially conscious behavior [41]. Culture explains people reactions to crisis; Kayser et al. [42] showed how the collectivist southern Indian culture shapes people response and coping with the 2004 Tsunami disaster. Cultural factors shape various health outcomes such as level of infection and control measures [37]. The results of this research contribute to this stream by showing that national culture explains the nation's vulnerability to pandemics.

Nations with different cultural background took different approaches to COVID-19 management [43]. Shabbir et al. ([44] p. 181) highlighted that inclusivity is key for efficient handling of COVID-19 across communities “… “social solidarity and care ethics nexus” can and should “travel” within and between societal strata.” Decision-makers can use the results to envisage the consumer groups more vulnerable to epidemics. The results suggest that two cultural factors (uncertainty avoidance and indulgence) are salient in explaining a nation's vulnerability. The culture's effect appears to dissipate on the longer term, when definite information about the epidemic is available, its repercussions are widely acknowledged, and a proper confinement policy is at place.

A comparison between the nations on a wide array of demographic, geographic, economic, and healthcare factors was performed, showing an indecisive role of these factors. While having a well-established healthcare system and a wealthier society should reduce vulnerability, such factors showed a modest role in the COVID-19 case. Importantly, poverty prevalence had a significant effect on vulnerability. Besides taking long-term measures to alleviate poverty, policymakers should focus on the poor in their communication and awareness campaigns. Further, they should provide proper healthcare access for the poor during the occurrence of an epidemic.

The results encourage policymakers and NGOs to consider culture to reduce vulnerability and alleviate the threats of epidemics at their infancy. Bhala et al. [45] emphasized the need to consider culture to improve health communication during COVID-19; for instance, showing sensitivity to racialized groups would improve communication effectiveness. Rathod [8] concludes, “… community engagement and culture-conscious policy can increase the effectiveness of any implementation and remain vitally important to the global struggle with the COVID-19 pandemic.”

The results suggest that certain nations, high on uncertainty avoidance and low on indulgence, are predominantly vulnerable. These consumer groups need comprehensive interventions, such as wide-ranging communication, behavioral intervention, and education [32]. Policymakers and NGOs should extend support to these groups upon an epidemic outbreak. Likewise, nations comprising societies with divergent cultures (e.g., Canada's French area, Southeast US, and Northeast US) would require preferential interventions. Further, cultural values can help optimize the creative of the communications, e.g., South Africa's *Ubuntu* or I am because we are, Senegal's *Nit nittay garabam* or the person is the remedy of the person, and Muslim countries' *Umma* or fraternity between all people as well as *Waman Ahyaha* or a person who saves one life is same as the person who saved all humanity.

This research has several limitations. First, the sample size is relatively small, which hindered performing additional analysis. Indeed, the sampling was performed for countries reporting COVID-19 cases at an early stage. Second, the results are obtained for one pandemic case (COVID-19). Future research should aim to verify the results on related pandemics. Third, the relation between culture and epidemics is vivid and not unidirectional. A body of research has focused on how infectious diseases shape cultural practices [13]. It argues that more infectious diseases a country faces over history lead to less individualism, extraversion and openness to experience, and more power distance (for a summary, see Table 1 in [46]). The latter authors built on this notion to develop an index reflecting the historical prevalence of epidemics for 230 nations and regions, with the aim of understanding the origin of cultural values and practices. As such, future research should study this dualism, e.g., the impact of COVID-19 on cultural shifts in the nations affected by it.

## Figures and Tables

**Table 1:** **H2** subhypotheses and their results.

National factor	Hypothesis, number	Coefficient *p* value	Finding
GDP per capita (PPP)	Negatively correlates with vulnerability, H2a	0.092	**Supported at 10% significance level**
Percent of population below poverty line	Positively correlates with vulnerability, H2b	0.028	**Supported**
Number of airports with paved runways	Positively correlates with vulnerability, H2c	0.251	Rejected
Literacy rate	Negatively correlates with vulnerability, H2d	0.363	Rejected
People using at least basic drinking water services (% of population)	Negatively correlates with vulnerability, H2e	0.496	Rejected
People using at least basic sanitation services (% of population)	Negatively correlates with vulnerability, H2f	0.138	Rejected
Health expenditure % GDP	Negatively correlates with vulnerability, H2g	0.221	Rejected
Health expenditure per capita (current US$)	Negatively correlates with vulnerability, H2h	0.417	Rejected
Cause of death, by communicable diseases and maternal, prenatal, and nutrition conditions (% of total)	Positively correlates with vulnerability, H2i	0.226	Rejected
Hospital beds (per 1,000 people)	Negatively correlates with vulnerability, H2j	0.033	Rejected (positive correlation was found)
Out-of-pocket healthcare expenditure per capita (current US$)	Positively correlates with vulnerability, H2k	0.435	Rejected
Physicians (per 1,000 people)	Positively correlates with vulnerability, H2l	0.277	Rejected
Mortality rate attributed to unsafe water, unsafe sanitation, and lack of hygiene (per 100,000 population)	Positively correlates with vulnerability, H2m	0.466	Rejected
Population ages 65 and above (% of total population)	Positively correlates with vulnerability, H2n	0.095	**Supported at 10% significance level**
Population growth (annual %)	Negatively correlates with vulnerability, H2o	0.069	**Supported at 10% significance level**
Universal healthcare (UHC) service coverage index	Negatively correlates with vulnerability, H2p	0.496	Rejected
Diabetes prevalence (% of population ages 20 to 79)	Positively correlates with vulnerability, H2q	0.201	Rejected
People practicing open defecation (% of population)	Positively correlates with vulnerability, H2r	0.209	Rejected
Geography: coastline in km	Positively correlates with vulnerability, H2s	0.358	Rejected
Urban population as % of total population	Positively correlates with vulnerability, H2t	0.296	Rejected
Population in major city in millions	Positively correlates with vulnerability, H2u	0.145	Rejected
Population density (people per sq. km of land area)	Positively correlates with vulnerability, H2v	0.162	Rejected
CO_2_ emissions (kg per 2011 PPP $ of GDP): carbon dioxide emissions are those stemming from the burning of fossil fuels and the manufacture of cement	Positively correlates with vulnerability, H2w	0.310	Rejected
PM2.5 air pollution, mean annual exposure (micrograms per cubic meter): population-weighted exposure to ambient PM2.5 pollution is defined as the average level of exposure of a nation's population to concentrations of suspended particles	Positively correlates with vulnerability, H2x	0.422	Rejected
Electricity production from coal sources (% of total): the inputs used to generate electricity. Coal refers to all coal and brown coal	Positively correlates with vulnerability, H2y	0.284	Rejected

**Table 2:** Correlation table of Hofstede's cultural factors with national vulnerability (*N* = 11).

Cultural factors	Power distance	Individualism	Masculinity	Uncertainty avoidance	Long-term orientation	Indulgence
Correlation coefficient	−0.359	0.060	0.179	0.657^*∗*^	0.329	−0.598^*∗*^
*p* value (one-sided)	0.139	0.430	0.299	0.014	0.161	0.026

## Data Availability

The secondary data collected to test the hypotheses are reported in Figure 1.
